# Development and validation of a nutrition-integrated nomogram for predicting 28-day mortality in sepsis patients

**DOI:** 10.3389/fnut.2025.1726151

**Published:** 2026-01-20

**Authors:** Yong-ling Yang, Yi-sheng Huang, Yu-long Bai, Xiao-xiang Huang, Zhao-yin Fu, Zhi-wei Huang

**Affiliations:** 1 Department of Critical Care Medicine, Tenth Affiliated Hospital of Guangxi Medical University, Qinzhou, China; 2 Department of Emergency, First Affiliated Hospital of Guangxi Medical University, Nanning, China

**Keywords:** biomarkers, machine learning algorithms, mortality, predictive model, sepsis

## Abstract

**Background:**

Sepsis is a life-threatening condition with high mortality, necessitating early risk stratification. This study aimed to develop and validate a predictive nomogram for 28-day mortality in sepsis patients incorporating machine learning-selected biomarkers.

**Methods:**

A total of 1,350 sepsis patients were retrospectively enrolled and divided into training (*n* = 944) and testing (*n* = 406) sets. LASSO and Random Forest (RF) algorithms were applied to screen key biomarkers associated with mortality. A logistic regression model was constructed using the selected features, and a nomogram was developed by integrating these biomarkers with APACHE II, SOFA score, and shock status. Model performance was evaluated by AUC, calibration, and decision curve analysis (DCA). External validation was performed in an independent cohort of 120 patients.

**Results:**

Six biomarkers consistently selected by both LASSO and RF were: procalcitonin (PCT), prognostic nutritional index (PNI), red blood cell count (RBC), platelet count (PLT), alanine aminotransferase (ALT), and indirect bilirubin (IBIL). Non-survivors exhibited significantly higher levels of PCT, ALT, and IBIL, and lower levels of RBC, PLT, and PNI compared to survivors (all *P* < 0.05). The logistic regression model demonstrated strong discrimination [AUC: 0.841 (95% CI: 0.814–0.868) in training set; 0.808 (95% CI: 0.769–0.847) in testing set]. The nomogram showed good calibration and favorable net clinical benefit across a wide range of threshold probabilities. In the external validation cohort, the model maintained excellent predictive performance with an AUC of 0.921 (95% CI: 0.876–0.966).

**Conclusion:**

We developed and validated a clinically useful nomogram incorporating nutrition-related biomarkers, particularly PNI, for predicting 28-day mortality in sepsis patients. The model demonstrates robust performance and highlights the importance of nutritional status in sepsis outcomes.

## Introduction

Sepsis, defined as a life-threatening organ dysfunction caused by a dysregulated host response to infection, remains a leading cause of morbidity and mortality in intensive care unit (ICU) worldwide (1). Despite advances in critical care, mortality rates remain high, particularly among patients with delayed recognition or suboptimal management. Early risk stratification is therefore essential to guide timely interventions, allocate resources efficiently, and improve patient outcomes. The Acute Physiology and Chronic Health Evaluation II (APACHE II) and Sequential Organ Failure Assessment (SOFA) scores are widely used for prognostication in sepsis. However, while these tools effectively capture physiological derangements and organ dysfunction, they may not fully reflect the underlying metabolic, immunological, and nutritional disturbances that profoundly influence host resilience and recovery (2). Emerging evidence underscores the critical interplay between inflammation, immune dysfunction, and malnutrition in the pathophysiology of sepsis. This can lead to catabolic exhaustion, impaired pathogen clearance, and increased susceptibility to secondary infections and multi-organ failure (3). In this context, the prognostic nutritional index (PNI) has gained recognition as a composite marker integrating nutritional status and immune function. Low PNI reflects both protein-energy malnutrition and lymphopenia-induced immunosuppression, both of which are independently associated with poor outcomes in critically ill patients (4, 5).

Notably, PNI has been shown to predict mortality in sepsis, yet it is often underutilized in routine clinical risk assessment models (6, 7). Recent advances in machine learning (ML) algorithms offer powerful tools for identifying complex, non-linear patterns in high-dimensional clinical data, enabling more accurate prediction of outcomes than traditional statistical methods (8). By combining ML-based feature selection with interpretable regression modeling, it is possible to develop robust predictive tools that balance accuracy with clinical usability (9, 10). However, few existing models incorporate nutrition-sensitive biomarkers, and even fewer have been translated into clinically intuitive nomograms, graphical calculators that allow bedside risk estimation, while undergoing rigorous external validation (11, 12). Therefore, this study aimed to develop and validate a novel, nutrition-informed predictive nomogram for 28-day mortality in adult sepsis patients. We integrated machine learning algorithms (LASSO and random forest) to identify key laboratory biomarkers, combined them with established severity scores (APACHE II, SOFA) and shock status, and validated the model both internally and externally.

## Materials and methods

### Study subjects

This was a retrospective cohort study conducted at two tertiary care centers: the First Affiliated Hospital and the Tenth Affiliated Hospital of Guangxi Medical University, from January 2024 to April 2025. The study protocol was reviewed and approved by the Institutional Review Board, and all procedures were conducted in accordance with the ethical principles outlined in the Declaration of Helsinki. Informed consent was waived due to the retrospective nature of the study.

### Inclusion and exclusion criteria

Patients were eligible for inclusion if they: (1) met the Third International Consensus Definitions for Sepsis and Septic Shock (Sepsis-3) criteria (13), defined as life-threatening organ dysfunction caused by a dysregulated host response to infection, with an increase in SOFA score ≥ 2 points; and (2) were aged 18 years or older. Exclusion criteria included: (1) pregnancy or lactation; (2) ICU length of stay < 24 h; (3) presence of advanced malignant disease or other terminal illnesses (life expectancy < 3 months); and (4) incomplete clinical or laboratory data that could not be reliably imputed.

### Collection of main clinical features

The clinical features were collected within 24 h of ICU admission. Collected variables included: demographic and clinical characteristics: age, sex, comorbidities (including diabetes, chronic kidney disease, cardiovascular disease), site of infection (including pulmonary, abdominal, urinary). Complete blood count (including white blood cell count, hemoglobin, platelets, lymphocytes), liver function tests (albumin, total protein, ALT, AST, bilirubin, cholinestersase), renal function (creatinine, BUN), and nutritional indicators (albumin or prealbumin). Severity of illness scores: APACHE II and SOFA scores, calculated based on the first 24 h of ICU admission. Interventions: use of mechanical ventilation, vasopressors, renal replacement therapy, and surgical interventions. The primary outcome was 28-day all-cause mortality, defined as death from any cause within 28 days of sepsis diagnosis. Calculation of NLR, PLR and PNI as follow: NLR = absolute neutrophil count/absolute lymphocyte count, PLR= absolute platelet count/lymphocyte count, PNI = 10 × serum albumin value + 0.005 × lymphocyte count (14). All data were cross-checked by two individuals, and missing values were imputed using multiple imputation methods. Missing data were handled using multiple imputation by chained equations (MICE) with 10 imputations, assuming missing at random (MAR), and pooled estimates were used in subsequent analyses.

### Biomarker selection using machine learning

To identify the most predictive laboratory biomarkers for 28-day mortality, we applied two machine learning algorithms: least absolute shrinkage and selection operator (LASSO) regression with binomial logistic loss function, using 10-fold cross-validation to select the optimal lambda (λ) that minimized the deviance. Random Forest (RF) with 500 trees, using the Gini index for variable importance ranking. The RF analysis was conducted with the following hyperparameters: number of estimators = 500, max_features = √*p* (where *p* is the number of input features), minimum samples per leaf (min_samples_leaf) = 5, and bootstrap sampling enabled. Hyperparameters were selected based on default settings after preliminary tuning showed minimal impact on feature ranking stability. The top 10 most important features were extracted based on mean decrease in accuracy. Variables consistently selected by both LASSO and RF were considered robust predictors and included in the subsequent multivariable logistic regression model. All machine learning analyses for biomarker selection were performed conducted using R software with the “glmnet” package and “randomForest” package.

### Nomogram model construction and evaluation

A multivariable logistic regression model was constructed using the selected biomarkers, along with established clinical predictors: APACHE II score, SOFA score, and presence of shock (defined as vasopressor requirement). The final model was visualized as a nomogram using the rms package (version 6.7-0) in R software (version 4.2.0). Model performance was evaluated in both internal (70% training cohort) and external (30% testing cohort) validation sets, with an independent external validation cohort from the Tenth Affiliated Hospital used to assess generalizability.

Discrimination was assessed using the area under the receiver operating characteristic curve (AUC), calculated with the pROC package. Calibration was evaluated using the calibration plot (observed vs. predicted probability), Brier score (lower values indicate better calibration), and Hosmer-Lemeshow goodness-of-fit test (*P* > 0.05 indicates good fit). Clinical utility was assessed via decision curve analysis (DCA), which evaluates net benefit across a range of threshold probabilities.

### Statistical methods

All statistical analyses were performed using R software (version 4.2.0). Categorical variables were presented as frequency (percentage) and compared using the chi-square (χ^2^) test or Fisher's exact test, as appropriate. Continuous variables were tested for normality using the Shapiro-Wilk test. Normally distributed data were expressed as mean ± standard deviation (SD) and compared using the independent samples *t*-test. Non-normally distributed data were presented as median [interquartile range, IQR: P25–P75] and compared using the Mann–Whitney *U* test. Comparison of predictive accuracy between different ROC was using Delong's test. A two-sided *P* value < 0.05 was considered statistically significant.

## Results

### Clinical characteristics of survivors and non-survivors

A total of 1,350 sepsis patients were included in this study, of whom 168 (12.4%) died during hospitalization and 1,182 (87.6%) survived. As shown in Table 1, non-survivors were significantly older than survivors (median age: 65 vs. 60 years, *P* < 0.05). Although the proportion of males was higher in the survivor group (821/1182, 69.5%) compared to the non-survivor group (124/168, 73.8%), no significant difference in sex distribution was observed between groups (*P* > 0.05). There were no significant differences in body mass index (BMI), history of smoking, or alcohol consumption between the two groups (*P* > 0.05). Non-survivors exhibited a significantly higher prevalence of shock, atrial fibrillation, and heart failure (*P* < 0.05 for all). With regard to treatment modalities, non-survivors were more likely to require ICU admission, mechanical ventilation, and vasopressor support, whereas corticosteroid use was less frequent in this group (*P* < 0.05). Non-survivors also had significantly higher APACHE II and SOFA scores compared to survivors (*P* < 0.001). No significant differences were observed in the distribution of infection sources, including pulmonary, urinary, hepatic, bloodstream, and other sites, between the two groups (*P* > 0.05). Laboratory analyses revealed that non-survivors had significantly higher levels of most routine blood tests, liver function markers, and renal function indicators, except for albumin, globulin, prealbumin, and hemoglobin, which were markedly lower in non-survivors (*P* < 0.05). Additionally, the NLR was significantly elevated in non-survivors, while the PLR and PNI were significantly reduced (*P* < 0.05). These findings collectively suggest that non-survivors exhibited a state of heightened systemic inflammation and severe nutritional impairment.

**Table 1:** Baseline characteristics of sepsis patients.

**Variables**	**Survival (*N =* 1,182)**	**Death (*N =* 168)**	***P* value**
Age	60.0 (52.0–70.0)	65.0 (50.5–78.0)	0.011
**Gender**			
Male	821 (69.5%)	124 (73.8%)	0.288
	Female	361 (30.5%)	44 (26.2%)
BMI	22.9 (20.4–25.7)	22.9 (20.0–26.0)	0.865
**Smoking**			
No	690 (58.4%)	104 (61.9%)	0.432
Yes	492 (41.6%)	64 (38.1%)	
**Alcohol**			
No	703 (59.5%)	108 (64.3%)	0.268
Yes	479 (40.5%)	60 (35.7%)	
**Shock**			
No	532 (45%)	7 (4.2%)	< 0.001
Yes	650 (55%)	161 (95.8%)	
**Heart failure**			
No	942 (79.7%)	106 (63.1%)	< 0.001
Yes	240 (20.3%)	62 (36.9%)	
**Atrial fibrillation**			
No	1,012 (85.6%)	124 (73.8%)	< 0.001
Yes	170 (14.4%)	44 (26.2%)	
**ARDS**			
No	1,046 (88.5%)	143 (85.1%)	0.256
Yes	136 (11.5%)	25 (14.9%)	
**ICU staying**			
No	407 (34.4%)	6 (3.6%)	< 0.001
Yes	775 (65.6%)	162 (96.4%)	
**Mechanical ventilation**			
No	576 (48.7%)	13 (7.7%)	< 0.001
Yes	606 (51.3%)	155 (92.3%)	
**Dialysis**			
No	1,084 (91.7%)	161 (95.8%)	0.087
Yes	98 (8.3%)	7 (4.2%)	
**Vasopressor**			
No	431 (36.5%)	1 (0.6%)	< 0.001
Yes	751 (63.5%)	167 (99.4%)	
**Corticosteroids**			
No	522 (44.2%)	90 (53.6%)	0.027
Yes	660 (55.8%)	78 (46.4%)	
APACHE II score	9.6 (7.1–12.4)	14.0 (9.8–19.3)	< 0.001
SOFA score	4.9 (4.0–5.7)	8.0 (6.4–9.6)	< 0.001
**Infected sites**			
Blood	237 (20.1%)	32 (19%)	0.830
Liver	242 (20.5%)	34 (20.2%)	
Lung	239 (20.2%)	33 (19.6%)	
Urinary	236 (20%)	40 (23.8%)	
Others	228 (19.3%)	29 (17.3%)	
**Laboratory test**			
WBC (×10^9^/L)	9.0 (6.3–13.3)	12.6 (7.0–19.9)	< 0.001
RBC (×10^9^/L)	2.8 (2.4–3.4)	2.4 (1.9–3.1)	< 0.001
HB (g/L)	80.0 (69.0–95.0)	69.3 (57.0–88.7)	< 0.001
PLT (×10^9^/L)	203.8 (99.0–322.9)	60.7 (27.0–114.8)	< 0.001
NEU (×10^9^/L)	6.8 (4.3–10.8)	11.3 (5.9–18.0)	< 0.001
LYM (×10^9^/L)	1.1 (0.7–1.7)	0.7 (0.3–1.1)	< 0.001
Monocyte (×10^9^/L)	0.6 (0.4–0.9)	0.4 (0.1–0.9)	< 0.001
TBil (μmoL/L)	12.2 (7.3–27.8)	35.0 (14.4–99.3)	< 0.001
DBIL (μmoL/L)	6.5 (3.6–19.4)	22.4 (9.1–68.8)	< 0.001
IBIL (μmoL/L)	5.1 (3.1–8.7)	9.2 (3.6–25.5)	< 0.001
Albumin (g/L)	31.2 (27.8–34.3)	28.7 (24.4–32.5)	< 0.001
Globulin (g/L)	28.9 (24.0–34.8)	23.5 (17.9–27.9)	< 0.001
A/G ratio	1.1 (0.9–1.3)	1.2 (1.0–1.7)	< 0.001
rGT (U/L)	73.0 (38.0–139.0)	55.0 (32.0–110.5)	0.021
TBA (μmoL/L)	6.4 (3.3–16.7)	16.5 (6.4–58.0)	< 0.001
AST (U/L)	34.0 (21.0–66.0)	158.5 (41.0–571.0)	< 0.001
ALT (U/L)	22.0 (10.0–45.0)	68.0 (14.5–324.0)	< 0.001
AST/ALT ratio	1.8 (1.1–3.1)	2.6 (1.6–4.3)	< 0.001
ALP (U/L)	119.0 (85.0–184.0)	126.5 (85.0–211.5)	0.374
Prealbumin (mg/L)	137.1 (87.1–207.8)	88.1 (60.8–126.7)	< 0.001
ChE (U/L)	3,435.5 (2,276.0–4,750.0)	3,021.0 (2,001.5–4,373.5)	0.047
PCT (μg/L)	1.0 (0.3–5.6)	6.9 (2.3–28.0)	< 0.001
PNI	360.0 (251.5–485.1)	206.7 (168.3–279.0)	< 0.001
PLR	171.6 (101.3–283.9)	82.5 (29.8–204.9)	< 0.001
NLR	6.1 (3.2–12.0)	15.8 (7.6–23.9)	< 0.001

WBC, white blood cell; RBC, red blood cell; HB, hemoglobin; PLT, platelet; NEU, neutrophil; LYM, lymphocyte; MONO, monocyte; NLR, neutrophil-to-lymphocyte ratio; PLR, platelet-to-lymphocyte ratio; PNI, prognostic nutritional index; TBiL, total bilirubin; DBiL, direct bilirubin; IBil, indirect bilirubin; ALB, albumin; GLO, globulin; GGT, gamma-glutamyltransferase; TBA, total bile acids; AST, aspartate aminotransferase; ALT, alanine aminotransferase; CHE, cholinesterase.

### Screening of key prognostic biomarkers using machine learning algorithms

**Figure 1:**
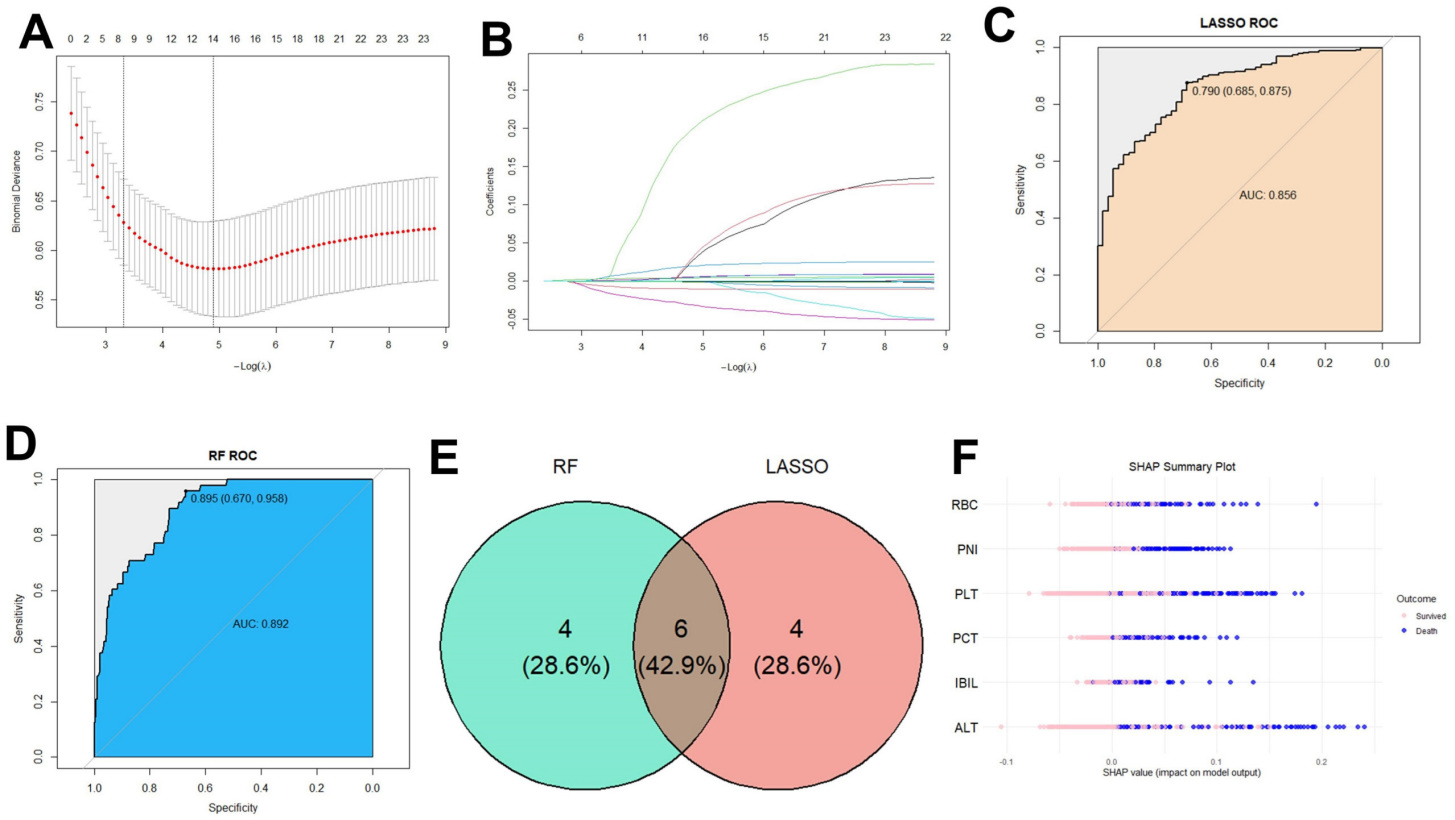
Screening of biomarkers associated with death using machine learning algorithms. **(A)** Selection of the penalty parameter λ using ten-fold cross validation. Figure shows two λ selection criteria in LASSO regression; **(B)** coefficient path of LASSO regression. Each colored line represents the coefficient trajectory of an independent variable as the penalty parameter λ changes; **(C)** ROC for the predictive value of the biomarkers on the death of sepsis patients by LASSO algorithms; **(D)** ROC for the predictive value of the biomarkers on the death of sepsis patients by RF algorithms; **(E)** venn plot for the common biomarkers from LASSO algorithms and RF algorithms; **(F)** SHAP summary plot for the biomarkers associated with the death of sepsis patients. The features are ranked based on the impact of their SHAP values on the outcome, with higher combined values indicating a higher risk of death within 28 days. Pink signifies high feature values, blue represents values close to the overall mean, and blue indicates low feature values.

Patients were randomly divided into a training set (*n* = 944) and a testing set (*n* = 406) at a 7:3 ratio. Two machine learning algorithms (LASSO and RF) were applied to screen 23 laboratory biomarkers for their association with mortality in the training cohort. LASSO regression identified 14 biomarkers with significant predictive value for mortality (Figures 1A–C). Similarly, RF analysis highlighted 10 top-ranking features associated with death (Figures 1D, E). By intersecting the results from both methods, six biomarkers were consistently selected, including PNI, platelet count (PLT), procalcitonin (PCT), alanine aminotransferase (ALT), red blood cell count (RBC), and indirect bilirubin (IBIL). These six markers were thus retained for subsequent model development. The SHAP summary plot illustrated the direction and magnitude of each biomarker contribution to the prediction outcome. SHAP summary plot showed that, the PNI, PLT, and RBC exerted the significantly negatively influence on the predicted mortality risk, with the mean |SHAP| value as 0.15; conversely, the PCT, ALT, and IBIL contributed positively to risk prediction, with the mean |SHAP| value as 0.12 (Figure 1F).

### Development and performance of the predictive model

**Figure 2:**
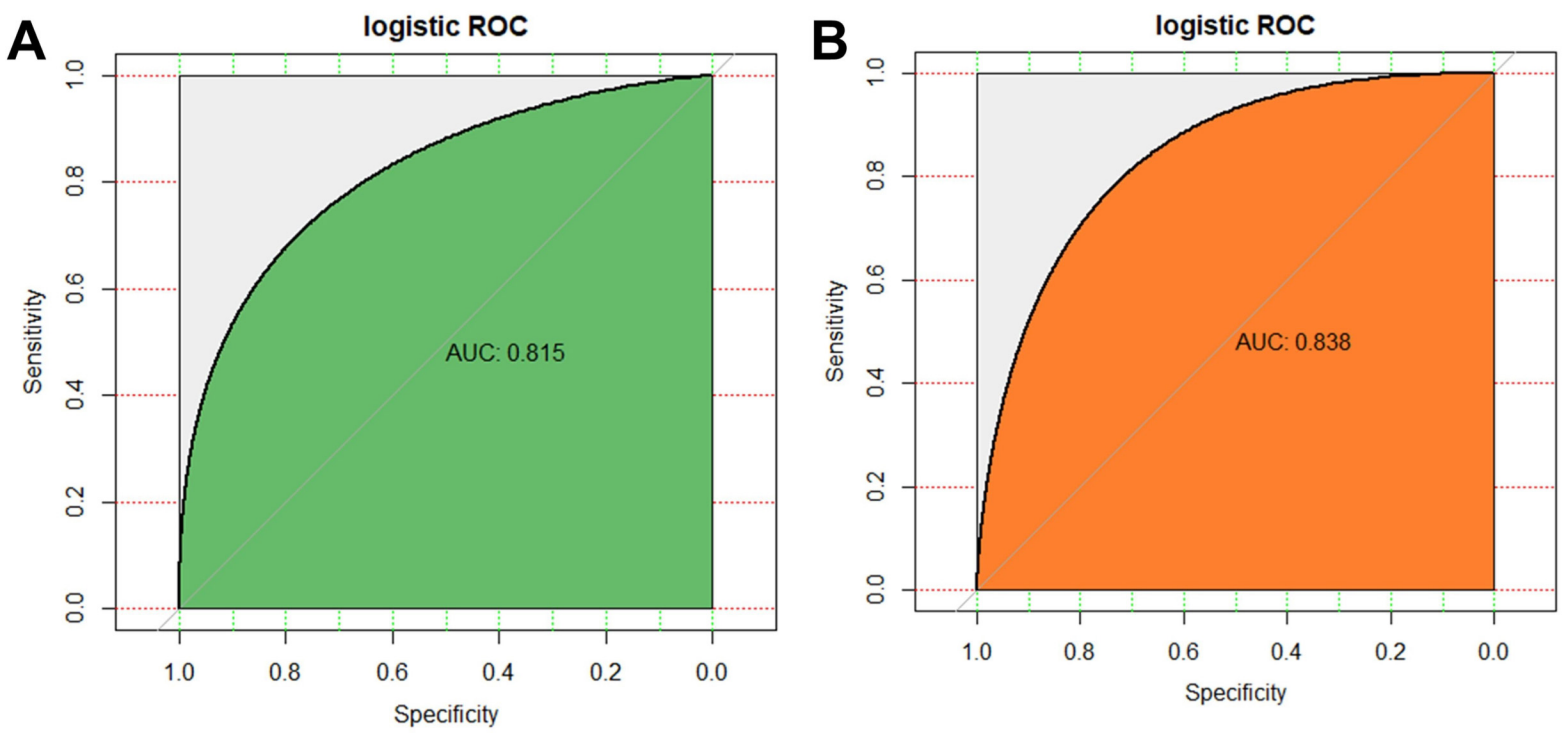
Predictive value of the model on the death of sepsis patients by logistic regression analysis. **(A)** ROC and AUC value in Training set; **(B)** ROC and AUC value in Testing set.

A logistic regression model was constructed using the six consensus biomarkers. Univariate logistic regression confirmed that each of the six biomarkers was significantly associated with mortality (all *P* < 0.05). Multivariate logistic regression further demonstrated that all six biomarkers were independent predictors of death in sepsis patients (Table 2). The resulting prediction model showed strong discriminative ability, with an AUC of 0.815 (95% CI: 0.712–0.870) in the training set and 0.838 (95% CI: 0.765–0.851) in the testing set (Figure 2). Using the Youden index, the optimal cut-off probability for predicting mortality was determined as 0.186. The full model equation and corresponding coefficients are detailed in Table 3.

**Table 2:** Logistic analysis of survival/death in patients with sepsis.

**Biomarkers**	**Training set OR (95%CI)**		**Testing set OR (95%CI)**	
	**OR (95%CI)**	***P*** **value**	**OR (95%CI)**	***P*** **value**
IBIL	1.02 (1.01–1.02)	0.001	1.00 (0.99–1.01)	0.403
ALT	1.00 (1.00–1.00)	0.001	1.00 (1.00–1.00)	0.011
PCT	1.03 (1.02–1.03)	0.001	1.01 (1.00–1.03)	0.016
RBC	0.55 (0.41–0.72)	0.001	0.47 (0.31–0.72)	0.001
PLT	0.99 (0.99–0.99)	0.001	0.99 (0.98–0.99)	0.001
PNI	0.99 (0.99–0.99)	0.001	0.99 (0.99–0.99)	0.001

**Table 3:** The predictive value of the model in training and testing set.

**Variables**	**Training set (95% CI)**	**Testing set (95% CI)**
AUC	0.821 (0.754–0.888)	0.845 (0.809–0.882)
Sensitivity	0.635 (0.75–0.865)	0.707 (0.784–0.862)
Specificity	0.757 (0.799–0.839)	0.734 (0.764–0.792)

### Nomogram construction and validation

**Figure 3:**
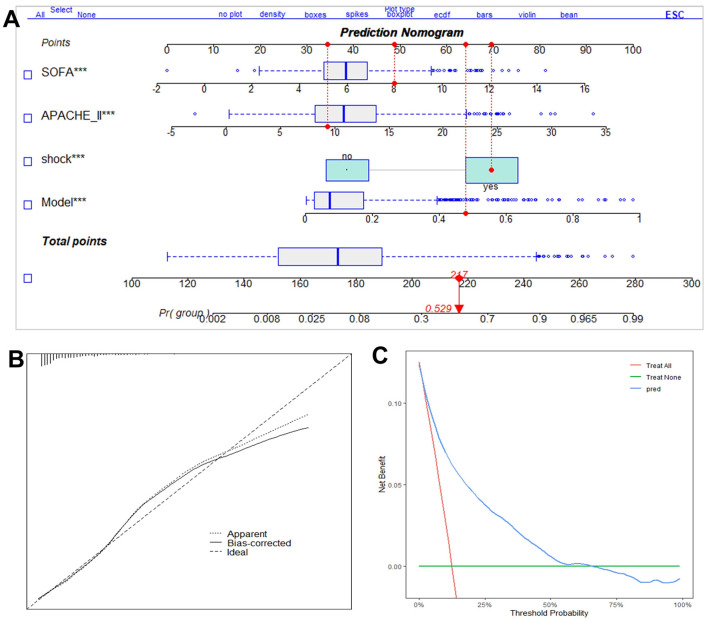
Construction of nomogram for the predictive model. **(A)** Nomogram for the predictive model and clinical parameters. Each independent predictor of Nomogram was mapped to a “points” value at the top of the nomogram, yielding a score within the range of 0–100. The total score was then used to accurately predict the outcome of sepsis patients, with higher scores indicating a higher risk of death; **(B)** calibration curve for the nomogram was used to evaluate the consistency between model predicted probabilities and actual observed probabilities; **(C)** decision curve analysis (DCA) assess the net benefit of the nomogram across different high-risk thresholds. ^***^p < 0.001.

To enhance clinical applicability, we developed a nomogram integrating the six-biomarker model with established clinical severity scores (APACHE II and SOFA) as well as the presence of shock, all well-recognized predictors of sepsis mortality (Figure 3A). Points assigned to each variable are summed to estimate the patient's probability of death. The nomogram demonstrated excellent discrimination, with a bootstrap-corrected C-index of 0.887 (95% CI: 0.863–0.911). Calibration analysis revealed good agreement between predicted and observed mortality probabilities (Figure 3B), supported by a low Brier score of 0.087. The Hosmer–Lemeshow test confirmed adequate model calibration (*P* = 0.624). DCA showed a positive net benefit across a wide range of threshold probabilities (Figure 3C), indicating robust clinical utility.

### External validation of the predictive model

**Figure 4:**
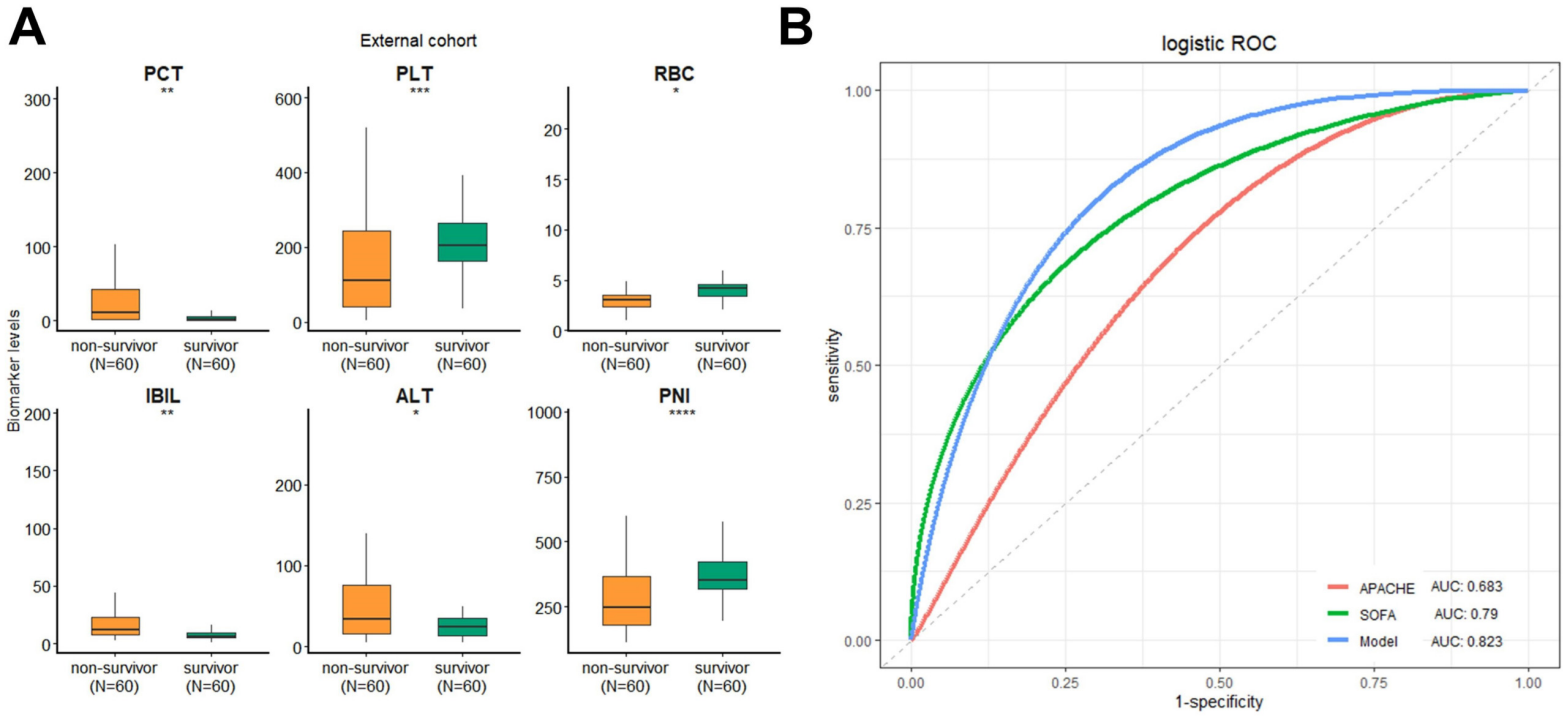
Validation of the predictive model by external cohort. **(A)** Comparison of the value of the six parameters in external cohort; **(B)** predictive value of the APACHE II score, SOFA score, and the predictive model. *P > 0.05, ** *P* > 0.01, *** *P* > 0.001, **** *P* > 0.0001.

To evaluate generalizability, the model was externally validated in an independent cohort of 120 sepsis patients from a different medical center, including 60 non-survivors and 60 survivors. The baseline characteristics are provided in Supplementary Table S1. Consistent with the primary cohort, non-survivors in the external set exhibited significantly higher levels of PCT, ALT, and IBIL, and lower levels of RBC, PLT, and PNI (*P* < 0.05; Figure 4A). When applied to this external cohort, the six-biomarker model achieved an AUC of 0.823 (95% CI: 0.758–0.907), and the predictive accuracy was better than APACHE II score (AUC: 0.683, 95% CI: 0.564–0.758) and SOFA score (AUC: 0.790, 95% CI: 0.731–0.886) (Figure 4B), indicating high predictive accuracy and strong cross-center reproducibility. Furthermore, the Delong's test showed that the predictive accuracy of the model was better than APACHE II score (*P* = 0.003), SOFA score (*P* = 0.035) and PNI (*P* = 0.016).

## Discussion

This study successfully developed and validated a clinically practical nomogram to predict mortality in sepsis patients by integrating a machine learning method to select laboratory biomarkers with established clinical severity scores. Among the 1,350 patients analyzed, non-survivors exhibited significantly higher disease severity as reflected by elevated APACHE II and SOFA scores, greater organ dysfunction, and more frequent ICU interventions. Additionally, pronounced inflammatory activation and malnutrition were observed in non-survivors. Using LASSO and RF algorithms on the training cohort, six biomarkers (PNI, PLT, PCT, ALT, RBC, and IBIL) were consistently identified as key predictors of mortality. The logistic regression model incorporating these markers achieved strong discriminative ability, with an AUC of 0.841 in the training set and 0.808 in the testing set. The final nomogram, further adjusted for APACHE II, SOFA, and shock status, demonstrated excellent calibration (Brier score: 0.087, Hosmer–Lemeshow *P* = 0.624) and a high C-index of 0.887. Importantly, external validation in an independent cohort of 120 patients confirmed exceptional predictive accuracy, highlighting the model robustness and generalizability.

The identified biomarkers (PNI, PLT, PCT, ALT, RBC, and IBIL) collectively reflect a convergence of nutritional status, systemic inflammation, organ dysfunction, and immune dysregulation, all critical determinants of mortality in sepsis. Previous studies have reported the association of PNI with the prognosis of sepsis, where lower PNI values indicate poor outcomes (15, 16). Thrombocytopenia (low PLT) and anemia (low RBC) suggest bone marrow suppression, microvascular consumption, and inflammatory hemolysis, all linked to poor prognosis (17, 18). Elevated PCT is a well-established marker of bacterial infection and sepsis severity, reflecting the magnitude of the inflammatory cascade (19, 20). Increased ALT and IBIL levels suggest hepatocellular stress and impaired bilirubin conjugation, often due to hypoperfusion or direct inflammatory injury. Lower IBIL levels have also been associated with poor outcomes in ICU patients with sepsis (21). Together, these biomarkers form a profile indicative of systemic inflammatory response compounded by metabolic and nutritional compromise.

Notably, the PNI emerged as a critical predictor. PNI integrates nutritional status and immune function, two interdependent factors frequently compromised in critically ill patients (22). Low PNI reflects malnutrition and immunosuppression, impairing the body's ability to combat infection and recover from organ injury (23). In this study, lower PNI was significantly associated with higher mortality, underscoring the detrimental impact of pre-existing or acute-on-chronic malnutrition in sepsis. Malnutrition leads to muscle wasting, weakened respiratory function, impaired wound healing, and diminished immune response, increasing susceptibility to secondary infections and organ failure (24). Furthermore, lymphopenia, a key component of PNI, is linked to T-cell exhaustion and immune paralysis in sepsis, contributing to persistent inflammation and poor outcomes (25). The consistent selection of PNI by both LASSO and random forest algorithms highlights its robust prognostic value. Given that nutritional interventions can modulate PNI, it may not only serve as a predictive biomarker but also as a potential target for early nutritional support, emphasizing the importance of integrating nutritional assessment into sepsis management protocols to improve survival.

This study offers significant clinical value by providing a readily applicable nomogram for early risk stratification of sepsis patients, integrating easily accessible laboratory markers, including the nutrition-immune index PNI. The model enables clinicians to identify high-risk patients promptly, facilitating timely interventions such as intensified monitoring, optimized antimicrobial therapy, and early nutritional support. The strong predictive performance, validated externally, supports its potential for use in diverse clinical settings. Notably, the inclusion of PNI underscores the critical role of nutritional status in sepsis outcomes, reinforcing the need for routine nutritional assessment and intervention in critical care. This aligns with the growing emphasis on personalized nutrition management. The nomogram's user-friendly format allows for quick risk estimation at the bedside, aiding clinical decision-making and potentially improving patient outcomes.

Despite previous studies (6, 7) have reported several models show good predictive ability on the death of sepsis, this study has several strengths. First, it employed machine learning algorithms to identify key biomarkers, enhancing feature selection reliability and reducing overfitting. The integration of these biomarkers into a logistic regression model and a clinically intuitive nomogram improves practical applicability. Second, the model demonstrated strong discriminative performance and excellent calibration in both internal and external validation cohorts, indicating high generalizability across different clinical settings. Third, the inclusion of PNI, a composite marker of nutritional and immune status, highlights the importance of nutritional factors in sepsis outcomes. The nomogram also incorporates established severity scores (APACHE II, SOFA) and shock status, increasing its clinical relevance. Importantly, our model is only presented in the form of a paper-based nomogram, which allows bedside risk estimation using routinely available data. Future work will aim to translate this tool into a digital format, potentially as a web-based calculator or an integrated module within the hospital information system, and therefore facilitate automated risk scoring at the point of care.

However, some limitations should be acknowledged. First, this study is retrospective and two-center in design, which may introduce selection bias. Although external validation was performed, the validation cohort was relatively small, and multi-center prospective studies are needed to further confirm the model reliability. Second, the dynamic changes in biomarker levels over time were not assessed, potentially limiting the model's ability to capture disease progression. Third, although our external validation cohort (*n* = 120) provides initial evidence of transportability, its modest size and single-center nature limit the precision of performance estimates and may not capture geographic or practice-related heterogeneity. Future multi-center prospective validation across diverse healthcare settings is essential before clinical deployment. Fourth, regarding the number of sepsis patients in external validation cohort, 60 (50%) patients died within 28 days. This balanced distribution was not artificially constructed; it reflects the high disease severity and mortality burden at this tertiary referral center during the study period. While such balance enhances statistical power for discrimination analysis, it may overestimate real-world prevalence and limit generalizability to lower-mortality settings. Finally, while nutritional status was partially evaluated through PNI, more comprehensive nutritional assessments (such as body composition, dietary intake) were not included, suggesting a direction for future refinement.

## Conclusion

This study developed and validated a robust predictive nomogram for 28-day mortality in sepsis patients by integrating key laboratory biomarkers identified through machine learning algorithms. The model demonstrated excellent discriminative accuracy, calibration, and clinical utility in both internal and external validation, highlighting its reliability and generalizability. Our results emphasize the integration of nutritional assessment into early sepsis management, supporting the paradigm shift toward precision nutrition in critical care.

## Data Availability

The original contributions presented in the study are included in the article/Supplementary material, further inquiries can be directed to the corresponding author.
