# Supplementary material for: Development and validation of a nutrition-integrated nomogram for predicting 28-day mortality in sepsis patients
Source: Front Nutr. 2026 Jan 20;12:1726151. doi: 10.3389/fnut.2025.1726151 (PMC12864124; doi:10.3389/fnut.2025.1726151)
Supplement: Supplementary file 1 [file Table_1.docx]

**Supplement file**

**Table S1 Baseline of external cohort**

| Variables | Value |
| --- | --- |
| Status |  |
| non-survivor | 60 (50.0%) |
| survivor | 60 (50.0%) |
| Age | 70.5 (62.5 - 80.5) |
| Gender |  |
| Male | 80 (66.7%) |
| Female | 40 (33.3%) |
| Surgery |  |
| No | 105 (87.5%) |
| Yes | 15 (12.5%) |
| Infected site |  |
| Abdomen | 10 (8.3%) |
| Respiratory tract | 81 (67.5%) |
| Urinary tract | 6 (5.0%) |
| Blood | 13 (10.8%) |
| Other | 10 (8.3%) |
| APACHE score | 26.0 (21.0 - 32.0) |
| SOFA score | 10.0 (7.0 - 13.0) |
| Treatment |  |
| Mechanical ventilation | 95 (79.2%) |
| Dialysis | 1 (0.8%) |
| Vasopressor | 24 (20.0%) |
| Bacterial type |  |
| Virus | 5 (4.2%) |
| Gram positive | 27 (22.5%) |
| Gram negative | 62 (51.7%) |
| Fungus | 24 (20.0%) |
| ScVo2 | 72.1 (65.8 - 75.2) |
| CPR | 59.1 (25.3 - 97.5) |
| PCT | 3.7 (0.6 - 19.2) |
| WBC | 13.5 (8.5 - 17.8) |
| NEU | 12.0 (7.0 - 15.4) |
| LYM | 0.7 (0.4 - 1.4) |
| PLT | 179.5 (97.5 - 263.5) |
| RBC | 3.5 (2.7 - 4.3) |
| HB | 94.0 (71.5 - 116.5) |
| ALB | 28.0 (24.4 - 32.4) |
| GLB | 26.6 (22.8 - 31.7) |
| TBIL | 14.3 (9.2 - 29.6) |
| DBIL | 7.0 (3.3 - 16.6) |
| IBIL | 8.2 (5.7 - 14.2) |
| ALT | 26.0 (15.0 - 50.0) |
| AST | 41.0 (23.5 - 70.5) |
| PNI | 327.2 (231.5 - 417.8) |
